# Effect of sex differences in remifentanil requirements for inhibiting the response to a CO_2_ pneumoperitoneum during propofol anesthesia: an up-and-down sequential allocation trial

**DOI:** 10.1186/s12871-020-0951-z

**Published:** 2020-02-03

**Authors:** Chengwei Yang, Yuanyuan Feng, Sheng Wang, Mingming Han, Song Wang, Fang Kang, Xiang Huang, Juan Li

**Affiliations:** 1 Department of Anesthesiology, The First Affiliated Hospital of USTC, Division of Life Sciences and Medicine, University of Science and Technology of China, Hefei, 230036 China; 2 Department of Hematology, The First Affiliated Hospital of USTC, Division of Life Sciences and Medicine, University of Science and Technology of China, Hefei, 230031 China

**Keywords:** Anesthesia, Pneumoperitoneum, Pharmacology, Remifentanil

## Abstract

**Background:**

A CO_2_ pneumoperitoneum during a laparoscopic procedure causes violent hemodynamic changes. However, the remifentanil required to inhibit the cardiovascular response to a CO_2_ pneumoperitoneum combined with propofol remains unknown. Moreover, the sex of the patient may influence the response to opioids, which can affect this requirement. The main objective of this study was to compare the required median effective concentration (EC_50_) of remifentanil for inhibiting the cardiovascular response to a CO_2_ pneumoperitoneum between female and male patients during propofol anesthesia.

**Methods:**

The current study is an up-and-down sequential allocation trial. Forty-six patients with American Society of Anesthesiologists physical status I or II, a body mass index 18 to 30 kg/m^2^, aged 20 to 60 years, and scheduled for laparoscopic surgery were enrolled. Induction of anesthesia was performed by target-controlled infusion. The effective effect-site concentration (Ce) of propofol was 4 μg/ml. The Ce of remifentanil was initially 4 ng/ml and then adjusted to a predetermined level after I-gel laryngeal mask airway insertion. The Ce of remifentanil for each patient was determined by the response of the previous patient using the modified Dixon “up-and-down” method. The first patient received remifentanil at 5.0 ng/ml Ce, and the step size between patients was 0.5 ng/ml.

**Results:**

Patients characteristics including age, body mass index, American Society of Anesthesiologists physical status, type of surgery and surgery duration, were comparable between male and female patients. The EC_50_ of remifentanil required to inhibit the response to a CO_2_ pneumoperitoneum based on the Dixon “up-and-down” method in women (4.17 ± 0.38 ng/ml) was significantly lower than that in men (5.00 ± 0.52 ng/ml) during propofol anesthesia (*P* = 0.01).

**Conclusions:**

The EC_50_ of remifentanil required to inhibit the response to a CO_2_ pneumoperitoneum was lower in women than in men during propofol anesthesia.

**Trial registration:**

The study was registered at http://www.chictr.org.cn (ChiCTR-IOR-17011906, 8th, July, 2017).

## Background

Currently, laparoscopic surgery is widely used due to its minimal invasiveness, low postoperative pain, short length of hospitalization and rapid postoperative recovery [1, 2]. However, a CO_2_ pneumoperitoneum during a laparoscopic procedure causes violent hemodynamic changes [3, 4]. Zou et al. [5] demonstrated that the response to a CO_2_ pneumoperitoneum was even stronger than that to the surgical incision.

A combination of remifentanil and propofol is commonly used for total intravenous anesthesia. Remifentanil has a very short infusion time-related half-life and is suitable for continuous infusion [6]. Moreover, it can inhibit the stress reaction effectively. The onset and recovery time of propofol are rapid, making sedation easy to control [7, 8]. Although there have been several studies investigating the median effective concentration (EC_50_) of remifentanil during propofol anesthesia in different situations [9–11], the remifentanil required to inhibit the cardiovascular response to a CO_2_ pneumoperitoneum combined with propofol remains unknown.

Moreover, the sex of the patient may influence the response to analgesic treatment with opioids, which can affect these requirements [12–14]. Men seem to need more opioids to achieve the same effect than women [15]. A recent study showed that the remifentanil requirements for the insertion of a laryngeal mask airway were higher in men than in women [16]. Accordingly, we hypothesized that the sex of the patient may affect the remifentanil required to inhibit the cardiovascular response to a CO_2_ pneumoperitoneum.

The main objective of this study was to compare the EC_50_ of remifentanil required to inhibit the cardiovascular response to a CO_2_ pneumoperitoneum between female and male patients during propofol anesthesia.

## Methods

The current study is an up-and-down sequential allocation trial. This study was approved by the Clinical Research Ethics Committee of Anhui Provincial Hospital (2016–110) and registered at http://www.chictr.org.cn (ChiCTR-IOR-17011906). Written informed consent was obtained from 46 patients undergoing elective laparoscopic surgery. The inclusion criteria were an age between 20 and 60 years old, an American Society of Anesthesiologists (ASA) physical status I or II and a body mass index (BMI) 18 to 30 kg/m^2^. The exclusion criteria included a history of cardiac, pulmonary, renal or liver diseases, alcohol or drug abuse, current use of vasoactive drugs, and recent use of any drugs known to affect the sympathetic adrenergic response.

All patients fasted routinely before surgery and received no premedication. Electrocardiograms, pulse oxygen saturation, end-tidal CO_2_ concentrations (EtCO_2_), and invasive radial arterial pressures were monitored. A bispectral index (BIS) monitor (BIS VISTATM monitor, Aspect Medical Systems, Norwood, MA) was used to monitor the depth of anesthesia.

Before anesthesia, 15 kg/ml lactated Ringer’s solution was administered and then maintained at a rate of 10 ml/kg/h. Patients were preoxygenated with 100% oxygen for 3 min. Induction of anesthesia was performed by a target-controlled infusion (TCI) pump (CP-730TCI; Inc., Beijing SLGO, China). The effective effect-site concentrations (Ce) of propofol (Marsh pharmacokinetic model) and remifentanil (Minto pharmacokinetic model) were 4 μg/ml and 4 ng/ml respectively. After loss of consciousness, 0.6 mg/kg rocuronium was injected intravenously, and then an I-gel laryngeal mask airway (LMA, size 3 for women, size 4 for men) was inserted. Mechanical ventilation was controlled with a tidal volume of 6–8 ml/kg and respiratory rate of 12–14 breaths per minute, maintaining the P_ET_CO_2_ within 35–45 mmHg. Three minutes after LMA insertion, the Ce of remifentanil was adjusted to a predetermined level. After maintaining the predetermined target Ce of remifentanil for at least 10 min, a CO_2_ pneumoperitoneum was established with a Veress insufflation needle. The pneumoperitoneum pressure of the machine (Inc., Stryker, America) was maintained at 14 mmHg, and the CO_2_ flow rate was 20 l per minute.

The Ce of remifentanil for each patient was determined by the response of the previous patient using the modified Dixon “up-and-down” method [17]. The first patient in each group received remifentanil at 5.0 ng/ml Ce, and the step size was 0.5 ng/ml. The response of patients to the CO_2_ pneumoperitoneum was determined by another anesthesiologist blinded to the remifentanil concentrations as either positive or negative. If the increase in the mean arterial pressure (MAP) or heart rate (HR) was more than 20% of its baseline, the response was defined as positive. In contrast, a negative response was defined as an increase in the MAP or HR of less than 20% of its baseline [5]. Patients’ MAP, HR and BIS values were recorded before induction, at baseline (defined as the average of 3 and 1 min measured values before the CO_2_ pneumoperitoneum) and 1 and 3 min after a stable pneumoperitoneum pressure was maintained. The increase in the MAP or HR was the difference between the average of the 1 and 3 min measured values after CO_2_ pneumoperitoneum and its baseline value. During this study, when the patient’s HR was less than 50 beats per minute, 0.5 mg atropine was injected intravenously. A bolus of 6–10 mg ephedrine was administered intravenously if the MAP was less than 50 mmHg. These patients were excluded from our study. The study was continued until 6 negative/positive crossover pairs had occurred. After finishing this study, intravenous administration of propofol and remifentanil was used to maintain the BIS between 40 and 60, and to ensure that the change in the MAP and HR did not exceed 20% of their baseline values. A TOF monitor (Veryark-TOF, Guangxi, China) was used to determine neuromuscular blockade. Rocuronium (0.15 mg/kg) was intravenously injected to maintain muscle relaxation when T_1_ twitch height reached 25% of the control.

Statistical analysis was performed using SPSS version 13.0 software (SPSS Inc., Chicago, IL). Data are expressed as the means ± standard deviations for continuous variables or the number (percentage) of patients. The EC_50_ of the remifentanil required to inhibit the cardiovascular response to a CO_2_ pneumoperitoneum in each group was determined by calculating the average of the midpoint dose of each pair of patients after 6 negative/positive crossover points were obtained. The “up-and-down” data were also analyzed by probit analysis [18, 19], deriving the EC_50_ and the 95% effective effect-site concentration (EC_95_) with their 95% confidence intervals (CIs). A *t* test was used to compare the EC_50_ values. Repeated measures analysis of variance was used to compare MAP, HR and BIS changes. All *P* values < 0.05 indicated significant differences.

## Results

Twenty-three male and 23 female patients were enrolled in this study. One female patient was excluded due to a MAP < 50 mmHg. One male patient was excluded due to an HR < 50 beats/min. Finally, 44 patients (22 male, 22 female) completed the study. The patients characteristics are shown in Table 1. Age, BMI, ASA physical status, type of surgery and surgery duration were comparable between the males and females. However, height and weight were significantly lower in the females.

**Table 1:** Patient Characteristics

	Male (*n* = 22)	Female (*n* = 22)	*P*
Age, years	38 ± 11	40 ± 10	0.56
Height, cm	172 ± 4	160 ± 5	<0.001
Weight, kg	69 ± 7	60 ± 10	0.001
BMI, kg/m^2^	23.4 ± 2.6	23.5 ± 3.7	0.94
ASA physical status (I/II)	18/4	20/2	0.39
Type of surgery (LA/LC)	15/7	14/8	0.75
Surgery duration	58.4 ± 13.4	57.3 ± 18.5	0.82

Values are presented as the means±standard or numbers. *BMI* Body mass index, *ASA* American society of anesthesiologists, *LA* Laparoscopic appendectomy, *LC* Laparoscopic cholecystectomy

**Fig. 1:**
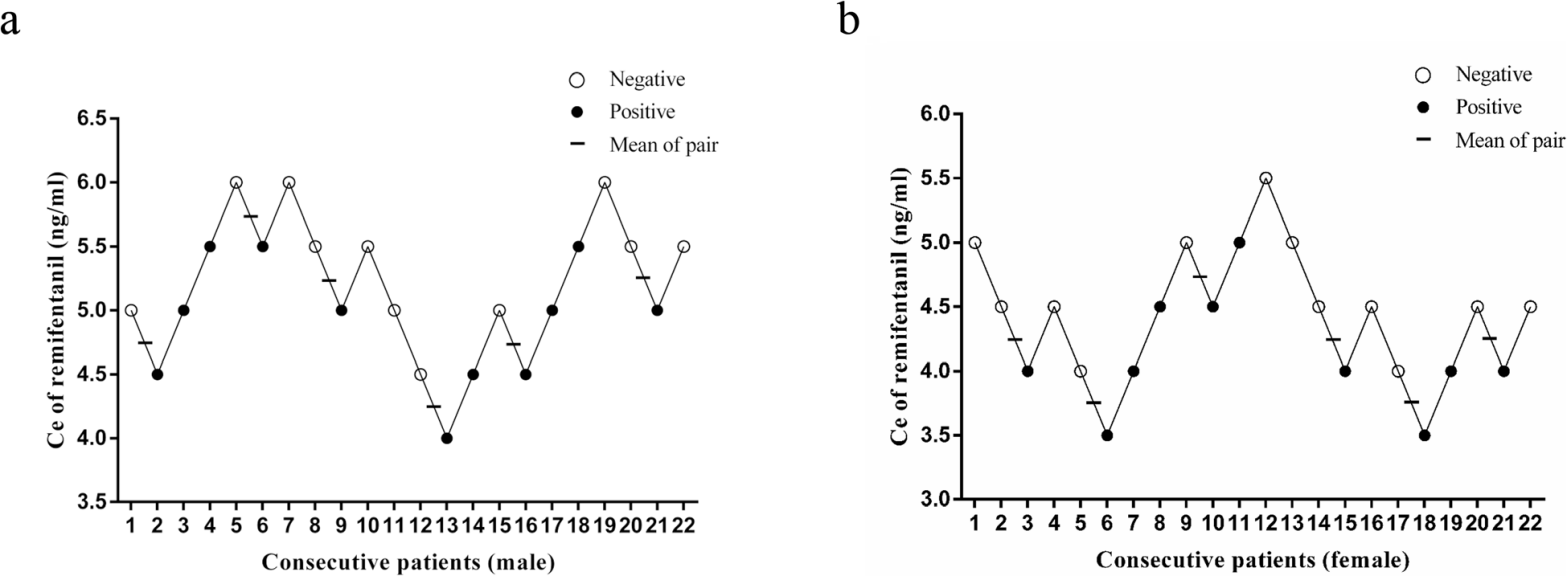
Assessment of negative or positive responses to inhibit a CO_2_ pneumoperitoneum under a predetermined Ce of remifentanil using the Dixon “up-and-down” method in 22 consecutive male patients (**a**) and 22 consecutive female patients (**b**). Horizontal bars represent crossover midpoints (negative to positive). The EC_50_ values of remifentanil required to inhibit the CO_2_ pneumoperitoneum in the male group and the female group were 5.00 ± 0.52 ng/ml and 4.17 ± 0.38 ng/ml, respectively. EC_50_, median effective concentration

The sequences for negative and positive responses to the CO_2_ pneumoperitoneum in the 2 groups are shown in Fig. 1. The EC_50_ of remifentanil required to inhibit a CO_2_ pneumoperitoneum based on the Dixon “up-and-down” method in women (4.17 ± 0.38 ng/ml) was significantly lower than that in men (5.00 ± 0.52 ng/ml) during propofol anesthesia (*P* = 0.01).

**Fig. 2:**
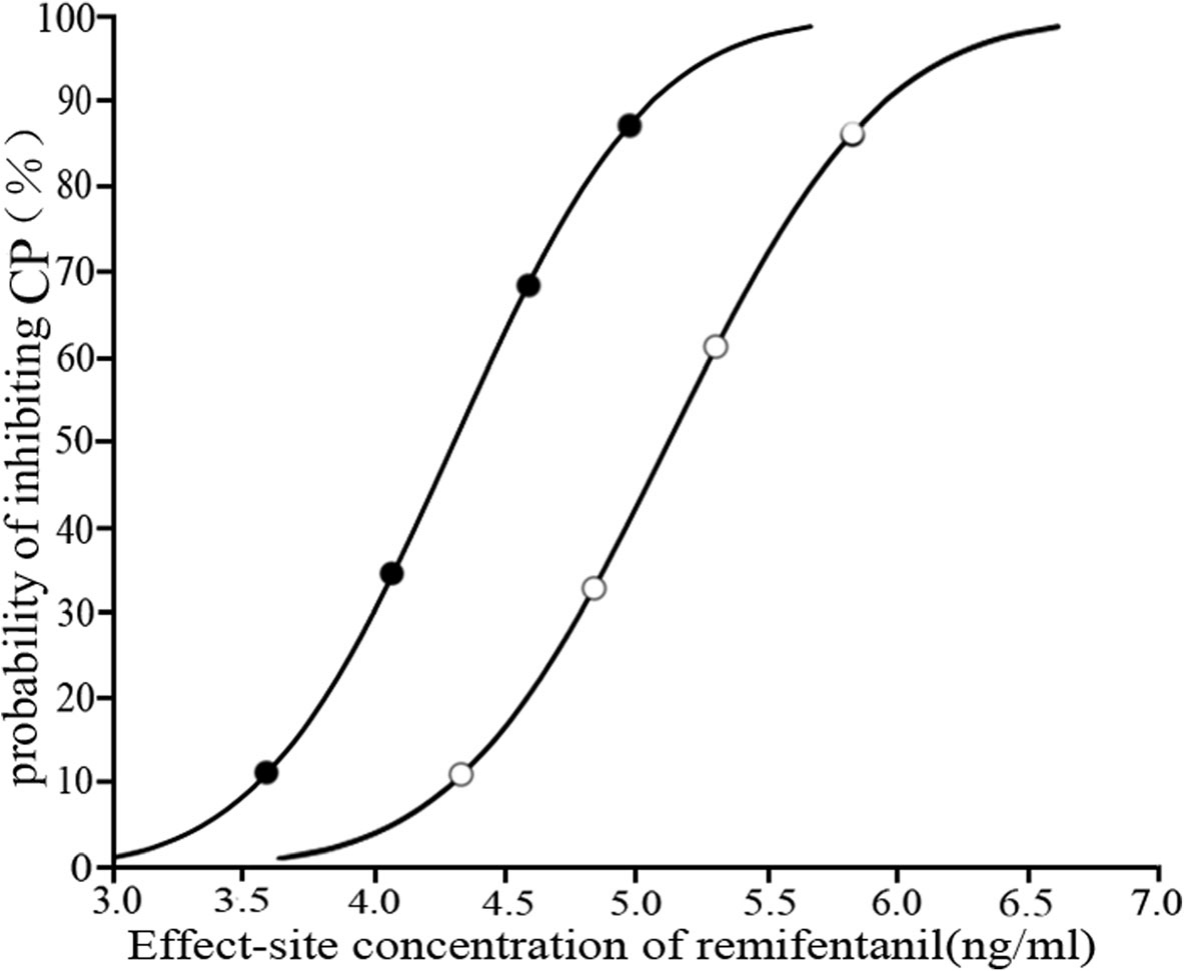
Effect-site concentration and response curves from the probit analysis in male (○) and female (●) patients. The EC_50_ and EC_95_ of remifentanil (95% confidence interval) required to inhibit the CO_2_ pneumoperitoneum were 4.30 (3.49–4.82) ng/ml and 5.27 (4.78–10.22) ng/ml in women and 5.16 (4.64–5.58) ng/ml and 6.19 (5.70–8.99) ng/ml in men, respectively. CP, CO_2_ pneumoperitoneum; EC_50_, median effective concentration; EC_95_, 95% effective effect-site concentration

From the probit analysis, the EC_50_ and EC_95_ of remifentanil (95% confidence interval [CI]) were 4.30 (3.49–4.82) ng/ml and 5.27 (4.78–10.22) ng/ml in women and 5.16 (4.64–5.58) ng/ml and 6.19 (5.70–8.99) ng/ml in men, respectively (Fig. 2).

The MAP, HR and BIS values over time are shown in Table 2. The hemodynamic and BIS data were not significantly different between the sexes.

**Table 2:** Hemodynamic profiles and BISs during the CO_2_ pneumoperitoneum

	Sex	Before induction	3 min before CP	1 min before CP	1 min after CP	3 min after CP
MAP, mmHg	Male	94 ± 11	73 ± 9	70 ± 8	87 ± 11	85 ± 11
	Female	92 ± 10	76 ± 11	70 ± 10	92 ± 13	88 ± 13
*P*		0.43	0.41	0.91	0.28	0.40
HR, beats/minute	Male	73 ± 8	61 ± 6	60 ± 6	68 ± 8	68 ± 8
	Female	76 ± 9	63 ± 7	60 ± 8	67 ± 11	66 ± 10
*P*		0.17	0.37	0.90	0.79	0.68
BIS	Male	96.3 ± 1.7	51.7 ± 6.4	49.7 ± 6.8	52.1 ± 6.5	51.8 ± 6.9
	Female	96.0 ± 1.4	52.1 ± 7.4	50.1 ± 7.7	53.0 ± 7.7	52.3 ± 8.1
*P*		0.51	0.83	0.85	0.69	0.83

Values are showed as the means±standard deviation. *MAP*, Mean arterial pressure; *HR* Heart rate, *BIS* Bispectral index, *CP* CO_2_ pneumoperitoneum

## Discussion

The present study demonstrated that the EC_50_ of remifentanil required to inhibit the cardiovascular response to a CO_2_ pneumoperitoneum in women (4.17 ± 0.38 ng/ml) was lower than that in men (5.00 ± 0.52 ng/ml) during propofol anesthesia.

The hemodynamic change induced by a CO_2_ pneumoperitoneum during laparoscopic surgery is a challenge to anesthesiologists [3, 20]. Remifentanil intravenously combined with general anesthesia provides stable hemodynamics during laparoscopic surgery [21]. Moreover, remifentanil effectively decreases the sevoflurane concentration to block the sympathetic adrenergic response to the CO_2_ pneumoperitoneum [5]. In this study, we were interested in exploring the EC50 of remifentanil required to inhibit the cardiovascular response to a CO_2_ pneumoperitoneum stimulus in both sexes during propofol anesthesia.

To the best of our knowledge, this is the first study investigating the EC_50_ of remifentanil required to inhibit the cardiovascular response to a CO_2_ pneumoperitoneum during propofol anesthesia. The EC_50_ of remifentanil required was higher than that measured by Albertin et al. (2.1 ng/ml) [9] or Wang et al. (3.09 ng/ml) [22]. The reason may be that a skin incision, which induces pain to the body surface and disappears fast, was used in their studies. This is consistent with Zou’s experiment [5]. The influence of CO_2_ pneumoperitoneum on the circulatory system is more complicated than that caused by a surgical incision. The increase in intra-abdominal pressure causes blood vessels to compress and reduces venous return, leading to a decrease in cardiac output. However, the increase in the partial pressure of CO_2_ in arterial blood, which may cause hypercapnia, can induce a sympathetic adrenergic response. As a result, the CO_2_ pneumoperitoneum causes an increase in blood pressure and heart rate [23, 24]. Furthermore, a continuous CO_2_ pneumoperitoneum distends the peritoneum and elicits a much stronger response than a skin incision. Accordingly, the required EC_50_ of remifentanil would be increased.

Increasing numbers of studies have focused on the sex differences in the response to anesthetics, especially opioids [12, 15, 16, 25]. In our study, the EC_50_ of remifentanil required to inhibit the cardiovascular response to a CO_2_ pneumoperitoneum was lower in women than in men during propofol anesthesia. There are some possible explanations for this result. First, Zubieta et al. demonstrated that premenopausal females have significantly higher mu-receptor binding potential than males in the cortical and subcortical areas [26]. Females may have a significantly greater response to mu opioid-receptor agonists than males [15, 27]. However, some studies have reported no sex differences in the analgesic responses to mu opioid- receptor agonists [28, 29]. Differences in the opioids, drug doses or pain models used may contribute to the different results. Second, previous studies have demonstrated that men have higher cortisol responses than women after exposure to acute real-life psychological stress or controlled laboratory stress tasks [30, 31]. However, sex differences in the hypothalamus–pituitary–adrenal axis responses to stress remain controversial [32, 33]. Moreover, whether sex differences exist in response to a CO_2_ pneumoperitoneum is unknown. Third, there is a sex difference in the activity of nonspecific esterase [34], which is responsible for metabolizing remifentanil. However, the specific esterase action on remifentanil remains unknown, and more research is needed.

According to a previous study, the EC_95_ (95%CI) of remifentanil required for successful LMA insertion in women and men was 3.38 (3.0–3.48) ng/ml and 3.94 (3.80–3.98) ng/ml, respectively, during propofol (Ce 3.5 μg/ml) anesthesia [16]. Therefore, the Ce of remifentanil was set at 4.0 ng/ml for LMA insertion. Furthermore, in our study, the heights and weights of the men were greater than those of the women. However, the influence of the differences in demographic data on our results may be small, as the Ce of remifentanil was calculated and adjusted by these covariates in the Minto model.

There are some limitations in our study. First, we did not take patients’ blood samples to measure the actual Ce of remifentanil. Instead, we calculated the remifentanil Ce using the Minto pharmacokinetic model, which has been widely used with acceptable levels of inaccuracy and bias in clinical settings [35]. Second, we did not perform arterial blood gas analysis to determine whether hypercarbia existed during the period of the CO_2_ pneumoperitoneum, which may have led to a sympathetic adrenergic response if the increased ventilation failed to compensate for the absorbed CO_2_. However, a previous study demonstrated that for changes in the P_ET_CO_2_ above 43 and below 26 mmHg, the mean arterial pressure increased and decreased, respectively [36]. In our study, we maintained the P_ET_CO_2_ within 35–45 mmHg to minimize the impact on blood pressure. Third, for safety considerations, we excluded patients with low MAPs and HRs, which may have resulted in an overestimation of the EC_50_ of remifentanil because these patients were possibly more sensitive to the drugs given. Lastly, the EC_95_ of remifentanil calculated by the “up-and-down” method may not be a reliable value [37], and further research may be needed for clinical practice.

## Conclusions

The EC_50_ of remifentanil required to inhibit the cardiovascular response to a CO_2_ pneumoperitoneum was lower in women than in men during propofol anesthesia using the modified Dixon “up-and-down” method. Patient sex should be taken into consideration for appropriate dosing when using remifentanil for laparoscopic surgery.

## Data Availability

The datasets generated and analyzed during the current study are not publicly available due to institutional restrictions but are available from the corresponding author on reasonable request.

## Abbreviations

ASA: American society of anesthesiologists
BIS: Bispectral index
BMI: Body mass index
Ce: Effect-site concentration
CP: CO_2_ pneumoperitoneum
EC_50_: Median effective concentration
EC_95_: 95% effective effect-site concentration
HR: Heart rate
LA: Laparoscopic appendectomy
LC: Laparoscopic cholecystectomy
LMA: Laryngeal mask airway
MAP: Mean arterial pressure
